# Diagnostic yield and impact of internal transcribed spacer sequencing from clinical specimens on antifungal management in patients with suspected fungal infection

**DOI:** 10.1128/spectrum.03964-25

**Published:** 2026-09-08

**Authors:** Visarut Techopitch, Pitak Santanirand, Angsana Phuphuakrat

**Affiliations:** 1 Department of Medicine, Faculty of Medicine Ramathibodi Hospital, Mahidol University 26687, Bangkok, Thailand; 2 Department of Pathology, Faculty of Medicine Ramathibodi Hospital, Mahidol University 26687, Bangkok, Thailand; London Health Sciences Centre, London, Ontario, Canada

**Keywords:** antifungal management, clinical specimens, diagnostic yield, impact, ITS sequencing

## Abstract

Internal transcribed spacer (ITS) sequencing has been increasingly used for the diagnosis of fungal infection. However, data on its real-world clinical utility are limited. We retrospectively evaluated the diagnostic yield from direct clinical specimens using the final diagnosis as the gold standard, and studied the impact of ITS sequencing on antifungal management in a university hospital with an unrestricted order setting. A total of 315 specimens were collected from 227 patients between November 2023 and April 2024. Of these, 59 (18.7%) were from fungal infection cases. ITS sequencing demonstrated a sensitivity of 35.6% and specificity of 96.9%. Agreement with fungal culture was 79.7% (κ = 0.583). In our setting, the median turnaround time of the positive results was 12 (12–14) days. ITS sequencing altered clinical management in only 2 patients (0.9%). The culture-negative but ITS sequencing-positive specimens mostly represented colonizers from bronchoalveolar lavage fluid. In an unrestricted setting, ITS sequencing showed low sensitivity and minimal impact on clinical decision-making. To maximize diagnostic benefit and utility, implementation of diagnostic stewardship is necessary.

**IMPORTANCE:**

This study provides a real-life evaluation of internal transcribed spacer (ITS) sequencing in an unrestricted clinical setting. We demonstrated fair sensitivity and minimal impact on antifungal alteration. We identified critical implementation barriers, including prolonged turnaround times and the frequent detection of colonizers, which fail to guide acute decision-making. These findings are vital for informing hospital policy and advocating for strict diagnostic stewardship to maximize clinical benefit.

## INTRODUCTION

Currently, the incidence and severity of fungal infections are increasing, posing a significant threat, particularly among immunocompromised patients (1). This vulnerable population includes recipients of solid organ or hematopoietic stem cell transplants, individuals with hematologic or solid malignancies, those with autoimmune diseases or primary immunodeficiencies, and patients receiving immunosuppressive drug therapy or living with human immunodeficiency virus. Late diagnosis and delayed treatment in these patients frequently contribute to unfavorable prognoses and poor treatment outcomes (2).

Traditionally, the diagnosis of fungal infections has relied on fundamental tools, including microscopic examination, conventional culture, and histopathology, which are correlated with clinical findings (3, 4). However, these methods are often hampered by limitations. Both microscopic examination and conventional culture exhibit lower sensitivity compared to molecular approaches, as their performance is dependent on the quality and quantity of the specimen, as well as the expertise of the laboratory personnel (5). Furthermore, conventional culture, although it remains the current standard for diagnosis of fungal infection, is often slow, resulting in delayed results (6).

Over the past decade, the development of molecular diagnostic methods has revolutionized fungal detection. Among these, internal transcribed spacer (ITS) sequencing has emerged as a powerful tool. ITS sequencing analyzes the highly variable non-coding regions, ITS1 and ITS2, located between the 18S and 28S rRNA genes, enabling the accurate identification and differentiation of fungal species (7–9). Molecular methods offer several key advantages: they can detect fastidious or slow-growing organisms that are difficult to culture and provide results more expeditiously than conventional techniques. Studies have demonstrated that ITS sequencing possesses high sensitivity, specificity, and a strong concordance with culture results from corresponding specimens, solidifying its important role in both diagnosis and species-level identification (10), although some medically important fungi require additional gene sequencing, e.g., the D1/D2 region of the 28S subunit, for species differentiation (11).

Despite the growing application of ITS sequencing, limited research data are available on its impact on clinical antifungal management. At our hospital, ITS sequencing is currently performed at the discretion of the attending physician without strict indication restrictions. This study aimed to contribute to the evidence base by addressing three key objectives: (i) evaluate the diagnostic yield of ITS sequencing in the diagnosis of fungal infections; (ii) assess the correlation between ITS sequencing results and culture results from corresponding specimens among patients with suspected fungal infections; and (iii) determine the impact of ITS sequencing on changes in the treatment of patients with fungal infections.

## MATERIALS AND METHODS

### Recruitment

A retrospective study was conducted among adult patients (≥18 years) at Ramathibodi Hospital (a 1,300-bed university hospital in Bangkok, Thailand) from November 2023 to April 2024. The study included patients whose attending physician requested ITS sequencing from a direct clinical specimen due to a suspicion of fungal infection. Patients were excluded if the ITS sequencing order lacked a corresponding fungal culture or if the order was a repeat request for follow-up purposes. ITS sequencing was available at the discretion of the attending physician during the study period. The study protocol received approval from the Human Research Ethics Committee at the Faculty of Medicine Ramathibodi Hospital, Mahidol University (approval no. COA.MURA2024/917).

Clinical, demographic, and microbiological data were collected retrospectively from patient medical records. Data collected included age, sex, immune status, provisional diagnosis, date of antifungal initiation, prior antifungal exposure, adjustments made to antifungal therapy following the ITS sequencing result, and the final diagnosis. The microbiological parameters collected included specimen type and quantity, results of Grocott’s methenamine silver (GMS) stain, conventional culture results, and the turnaround time (TAT) for the ITS sequencing result.

### Definitions

The ITS sequencing-positive group included the results that identified a fungal genus/species. The ITS sequencing-negative group was defined as the sequencing results that failed to identify a fungal genus/species.

ITS sequencing was considered to have an impact on antifungal management when the ITS sequencing result led to a documented change in antifungal therapy (e.g., initiation, adaptation/modification, or discontinuation/withholding) starting from the date the result was reported. Fungal infections included those caused by yeasts, molds, and dimorphic fungi.

Fungal infection as the cause of disease was established by the attending physician’s final diagnosis, which was based on the patient’s clinical condition, microbiological and/or molecular results, and the response to antifungal treatment. Non-fungal infection was determined by the attending physician’s final diagnosis, encompassing bacterial and viral infections, as well as non-infectious conditions such as autoimmune diseases, inflammatory diseases, malignancies, or adverse drug events.

Community-acquired infection was defined as an infection present upon hospital admission or occurring within the first 48 h of admission. An infection acquired after 48 h of hospitalization was categorized as a healthcare-associated infection.

### ITS sequencing procedure

#### Specimen processing and DNA extraction

For fluid specimens (e.g., bronchoalveolar lavage fluid and abscess fluid), 1.5 mL was collected and centrifuged at 15,000 × g for 5 minutes. After removing 1.1 mL of the clear supernatant, the remaining 400 µL was used for DNA extraction. If the specimen volume was less than 1.5 mL, 400 µL was directly used without centrifugation. For tissue specimens, approximately 10 mg of tissue was homogenized in 400 µL of tissue lysis buffer and 20 µL of proteinase K (20 mg/mL; Precision System Science, Chiba, Japan), followed by overnight incubation at 56°C. The lysates were then centrifuged, and 400 µL of the resulting supernatant was used for DNA extraction. All DNA extractions were performed using the magLEAD-12gC automated extraction system (Precision System Science).

#### PCR amplification and sequencing

A pair of primers (ITS1 and ITS4) was used for PCR reactions. The primers (12) used were ITS1 (forward primer) with a sequence of TCC GTA GGT GAA CCT GCG G and ITS4 (reverse primer) with a sequence of TCC TCC GCT TAT TGA TAT GC (Bioneer, Daejeon, Republic of Korea). The ITS1 and ITS2 regions were amplified by PCR, and the resulting products were analyzed by agarose gel electrophoresis, where DNA bands of expected sizes were visualized under UV light. The amplified PCR products were purified using the ExoSAP-IT PCR Product Cleaning Reagent (Thermo Fisher Scientific, Waltham, MA, USA). Purified products were stored at 4°C and transported via air courier to Bioneer Corporation (Daejeon, Republic of Korea) at least twice a week. Sanger sequencing was performed using capillary electrophoresis, and the resulting sequences were analyzed against the National Center for Biotechnology Information database using BLASTn to identify the fungal species. The analytical sensitivity was 10^4^ CFU/mL. Five identified genera and/or species with the highest identity score were reported to clinicians.

### Statistical analysis

Continuous variables were presented as mean ± standard deviation or median (interquartile range, IQR). Categorical variables were summarized using frequency and percentage. Continuous variables were compared using the Mann-Whitney *U* test or *t*-test, while categorical variables were compared using Fisher’s exact test or the χ^2^ test. A *P* value of <0.05 was considered statistically significant.

The diagnostic performance of ITS sequencing was assessed by calculating sensitivity and specificity, using the attending physician’s final diagnosis as the gold standard. The κ coefficient was employed to evaluate the level of agreement between ITS sequencing and conventional culture results. All statistical analyses were performed using STATA 18.0 (StataCorp, College Station, TX, USA).

## RESULTS

### Patient characteristics

During the study period, a total of 315 specimens were requested for ITS sequencing from 227 patients. The median age of the patients was 61.6 (50.1–72.7) years, with 100 (44.1%) being male. Nearly half of the cohort (44.9%) was immunocompromised. The causes of immunocompromise included solid malignancy (*n* = 29), hematologic malignancy (*n* = 23), autoimmune disease (*n* = 22), solid organ transplant (*n* = 14), adult-onset immunodeficiency syndrome (*n* = 7), acquired immunodeficiency syndrome (AIDS, *n* = 5), and hematopoietic stem cell transplantation (HSCT, *n* = 2). The most common provisional diagnoses were skin and soft tissue infection (33.0%) and invasive pulmonary fungal infection (28.2%). Other diagnoses included osteomyelitis/spondylodiscitis (7.1%), septic arthritis (6.6%), prosthetic joint infection (5.7%), endophthalmitis/uveitis (3.5%), and invasive fungal sinusitis (3.1%).

In this cohort, specimens from 22 patients had positive ITS sequencing (Table S1). Patients who had positive ITS sequencing were found to be older and were more frequently associated with healthcare-associated infections (Table 1). A statistically significant difference was observed in the proportions of provisional diagnoses between the ITS sequencing-positive and -negative groups (*P* = 0.042) (Table 1). The most common provisional diagnosis in the ITS-positive group was invasive pulmonary fungal infection (14/22, 63.6%), followed by skin and soft tissue infection (3/22, 13.6%) and invasive fungal sinusitis (2/22, 9.1%). Conversely, the ITS-negative group most frequently presented with skin and soft tissue infection (72/205, 35.1%), invasive pulmonary fungal infection (50/205, 24.4%), osteomyelitis/spondylodiscitis and septic arthritis (15/205, 7.3% each).

**TABLE 1:** Clinical characteristics and demographics of the patients

Parameter	ITS sequencing positive (*n* = 22)	ITS sequencing negative (*n* = 205)	*P* value
Median (IQR) age, years	67.0 (56.0–79.2)	61.0 (49.0–72.1)	0.026
Male, *n* (%)	11 (50.0)	89 (43.4)	0.554
Immune status, *n* (%)			0.160
Immunocompetent	9 (40.9)	116 (56.6)	
Immunocompromised	13 (59.1)	89 (43.4)	
Infection, *n* (%)			0.003
Community-associated	15 (68.2)	189 (92.2)	
Healthcare-associated	7 (31.8)	16 (7.8)	
Prior antifungal exposure, *n* (%)			0.407
No prior antifungal	19 (86.4)	189 (92.2)	
Within 90 days	3 (13.6)	16 (7.8)	
Specimen, *n* (%) (*n* = 315)			0.176
Fluid	15 (51.7)	111 (38.8)	
Tissue	14 (48.3)	175 (61.2)	
GMS positive for fungus^*a*^, *n* (%)	10 (38.5)	4 (1.9)	<0.001
Fungal culture positive^*b*^, *n* (%)	22 (75.9)	22 (7.7)	<0.001
Median (IQR) turnaround time, days	12 (12–14)	2 (1, 2)	<0.001
Provisional diagnosis, *n* (%)			0.042
Invasive pulmonary fungal infection	14 (63.6)	50 (24.4)	
Skin and soft tissue infection	3 (13.6)	72 (35.1)	
Invasive fungal sinusitis	2 (9.1)	5 (2.4)	
Osteomyelitis/spondylodiscitis	1 (4.6)	15 (7.3)	
Keratitis/scleritis	1 (4.6)	4 (2.0)	
Brain abscess	1 (4.6)	2 (1.0)	
Septic arthritis	0 (0.0)	15 (7.3)	
Liver abscess	0 (0.0)	6 (2.9)	
Prosthetic joint infection	0 (0.0)	13 (6.3)	
Lymphadenopathy	0 (0.0)	5 (2.4)	
Intraabdominal collection/abscess	0 (0.0)	4 (2.0)	
Endophthalmitis/uveitis	0 (0.0)	8 (3.9)	
Meningitis	0 (0.0)	3 (1.5)	
Endocarditis	0 (0.0)	1 (0.5)	
Splenic abscess	0 (0.0)	1 (0.5)	
Pancreatic abscess	0 (0.0)	1 (0.5)	

^*a*^ GMS, total 239 specimens; ITS sequencing positive, 26 specimens; ITS sequencing negative, 213 specimens.

^*b*^ Fungal culture, total 315 specimens; ITS sequencing positive, 29 specimens; ITS sequencing negative, 286 specimens.

Of the total 315 requested specimens, 40.0% (*n* = 126) were fluid samples and 60.0% (*n* = 189) were tissue samples. The most common fluid types were bronchoalveolar lavage (BAL) fluid (*n* = 59), abscess fluid (*n* = 21), and synovial fluid (*n* = 20). The proportions of positive GMS staining for fungus and positive fungal culture results were significantly higher in the ITS sequencing-positive group (*P* < 0.001, both). The TAT of the ITS sequencing results was significantly longer for the ITS sequencing-positive group, at 12 (12–14) days, compared to 2 (1, 2) days for the negative group (*P* < 0.001).

Overall, most patients (*n* = 208, 91.6%) were not receiving antifungal therapy when the specimen was collected. When comparing the ITS sequencing groups, there was no significant difference in the proportion of patients exposed to antifungals between the positive (8.3%) and negative (13.64%) groups (*P* = 0.407). Additionally, the specimen type (fluid vs. tissue) did not differ significantly between the positive and negative sequencing outcomes (*P* = 0.176).

### ITS sequencing results

Of the total specimens collected, 59 (18.7%) came from 39 patients with a final diagnosis of fungal infection, while the remaining 256 (81.3%) specimens from 188 patients were associated with a non-fungal infection diagnosis (Table 2). ITS sequencing yielded a sensitivity of 35.6% [21/59] and a 96.9% [248/256] specificity.

**TABLE 2:** Comparison of ITS sequencing results categorized by final diagnoses of fungal vs non-fungal infections

ITS sequencing results	Fungal infection *N* (%)	Non-fungal infection *N* (%)	Total *N* (%)
Positive	21 (35.6)	8 (3.2)	29 (9.2)
Negative	38 (64.4)	248 (96.9)	286 (90.8)
Total	59	256	315

When evaluating the fungal infection cases, ITS sequencing results were compared with fungal culture (Table 3). Overall, the two methods showed agreement at 79.7 (κ coefficient, 0.583; *P* < 0.001). In culture-negative fungal infection cases, 9.4% (3/32) of specimens were ITS sequencing-positive, which represented 5.1% of the total fungal infection specimens. Conversely, in culture-positive fungal infection cases, 33.3% (9/27) of specimens were ITS sequencing-negative, which represented 15.3% of the total fungal infection specimens.

**TABLE 3:** Comparison of fungal culture and ITS sequencing results among patients with a final diagnosis of fungal infection

ITS sequencing results	Culture positive *n* (%)	Culture negative *n* (%)	Total *N* (%)
Positive	18 (66.7)	3 (9.4)	21 (35.6)
Negative	9 (33.3)	29 (90.6)	38 (64.4)
Total	27	32	59

As shown in Table 4, 126 of 315 specimens (40.0%) were fluid samples. Among these, ITS sequencing yielded positive results in 15 of 126 samples (11.9%). All ITS sequencing-positive results were obtained from fluid specimens with a volume >1.5 mL. For tissue samples, 14 (7.4%) were positive by ITS sequencing. However, the proportion of ITS sequencing-positive results in tissue was not significantly different between tissue weights higher or lower than 10 mg.

**TABLE 4:** Characteristics of the specimens in this study

Specimen *n* (%)	ITS sequencing positive (*n* = 29)	ITS sequencing negative (*n* = 286)	*P* value
Fluid	15 (51.7)	111 (38.8)	0.176
Fluid volume			0.021
≤1.5 mL	0 (0.0)	30 (27.0)	
>1.5 mL	15 (100.0)	81 (73.0)	
Fluid type			0.007
Bronchoalveolar lavage	15 (100.0)	44 (39.6)	
Abscess	0 (0.0)	21 (18.9)	
Synovial fluid	0 (0.0)	20 (18.0)	
Vitreous humor	0 (0.0)	10 (9.0)	
CSF	0 (0.0)	5 (4.5)	
Pleural effusion	0 (0.0)	4 (3.6)	
Ascites	0 (0.0)	4 (3.6)	
Others	0 (0.0)	3 (2.7)	
Tissue	14 (48.3)	175 (61.2)	0.176
Tissue weight			0.508
Tissue ≤ 10 mg	1 (7.1)	8 (4.6)	
Tissue > 10 mg	13 (92.9)	167 (95.4)	
Tissue type			>0.999
Soft tissue	13 (92.9)	156 (89.1)	
Bone	1 (7.1)	19 (10.9)	

### Impact on antifungal management

Of the 315 specimens from 227 cases, ITS sequencing had a direct impact on antifungal management in only 2 of 227 total patients (0.9%), representing 5.1% (2/39) of the patients with a final fungal infection diagnosis. The two clinically significant cases were: (i) identification of *Phaeoacremonium* spp., which led to surgical debridement as the primary treatment. No antifungal drug was required due to adequate debridement; (ii) identification of *Chrysocorona lucknowensis* in a brain abscess, which prompted the adjustment of the antifungal regimen by giving the combination of amphotericin B and voriconazole (previously reported in [13]).

Among fluid specimens, only BAL yielded ITS-positive results. However, most of these detected *Candida* spp., a common respiratory colonizer, and consequently did not prompt antifungal management (Table S1).

A total of seven specimens were culture-negative but ITS sequencing-positive. However, none of these results led to a change in antifungal management. In six of these cases, the ITS sequencing result was ultimately determined to represent colonization or non-fungal infection. The pathogens identified in these six cases were *Cladosporium cladosporioides* (in disseminated pulmonary tuberculosis/probable invasive pulmonary aspergillosis), *Candida glabrata* (in probable invasive pulmonary aspergillosis), *Candida albicans* (in amiodarone-induced pneumonitis), *Candida glabrata* (in recurrent *Pseudomonas aeruginosa* pneumonia), *Candida nivariensis* (in chronic osteomyelitis of the hip), and *Cladosporium* spp. (in chronic infection of the forearm). The seventh specimen identified *Exserohilum* spp. in a case of invasive fungal rhinosinusitis from *Aspergillus* spp. and *Exserohilum* spp. However, this ITS result did not alter the existing treatment, as empirical antifungal treatment with voriconazole had already been initiated.

## DISCUSSION

Our findings demonstrate the clinical utility of ITS sequencing in an unrestricted testing environment, identifying fungi in 35.6% of confirmed fungal infection cases. However, from a diagnostic stewardship perspective, the clinical impact of ITS sequencing was limited. The sequencing had a direct and demonstrable impact on antifungal management in only two cases (5.1%).

The overall sensitivity of ITS sequencing in our study was substantially lower (35.6%) than the 87.7% to 95.6% reported elsewhere (14, 15). This is likely due to the gold standard used in the different studies. When we used fungal culture as the gold standard, the sensitivity of ITS sequencing in this data set was 75.9%, which was slightly lower than that reported in other studies (15, 16). The higher proportion of ITS sequencing-positive in specimens with GMS and/or culture positive for fungus suggests that when the fungal burden is high, ITS sequencing reliably detects the pathogen. Additionally, our data emphasize the importance of specimen quantity, with large specimens, especially fluid specimens, showing a significantly higher rate of positive ITS results. Adequate specimen collection is therefore a prerequisite for successful molecular diagnosis (8). Lau A et al., recommended that the panfungal PCR assay should be performed only in cases where fungal hyphae are visualized and where either the culture result is negative or culture is not performed (5).

The concordance of positive results between fungal culture and ITS sequencing was predominantly observed in invasive pulmonary fungal infection. However, most of the ITS sequencing results from the BAL fluid were interpreted as colonization. This highlights a critical diagnostic challenge: in respiratory samples, a positive ITS result indicates the presence of fungal DNA but does not reliably distinguish between true infection and colonization or contamination. A patient’s history and clinical findings must be considered when making a diagnosis.

Our findings of the small impact on antifungal management are starkly lower than those reported in the literature, such as Guenter S et al., who found that ITS sequencing by next-generation sequencing influenced management, including confirmation, changed, or support for withholding of antifungal treatment, in 90% of cases (17). However, only 15% resulted in an adjustment of antifungal therapy. Lieberman JA and coworkers reported that broad-range fungal PCR in cases with suspected fungal sinusitis identified pathogens missed by culture in 14.9% of cases and influenced clinical management in 16.7% of cases (18). This discrepancy partly resulted from differences in clinical settings and sequencing methods among the studies. Sanger sequencing has inherent sensitivity limitations and performs optimally only in monomicrobial samples. Next-generation sequencing, however, can analyze mixed or polymicrobial clinical samples with higher sensitivity (19).

Also, a significant barrier to clinical utility in our study was the long TAT for sequencing results. For many patients, empirical antifungal treatment was already completed or adjusted before the definitive ITS result became available. A late test, regardless of its accuracy, offers minimal clinical benefit, underscoring that improvements in laboratory processing speed are essential to maximize the clinical value of this molecular tool. In settings with rapid TAT of the results, positive clinical outcomes have been described (20).

The small number of specimens that were ITS-positive but culture-negative suggests that this technique offers limited diagnostic value beyond conventional microbiology. Moreover, in our study, most of these discordant results were attributed to colonization or contamination, highlighting the necessity for stricter ordering policies. Consequently, we discourage ITS sequencing of BAL fluid in our setting, as it predominantly detects *Candida* spp., a common colonizer.

A strength of this study was the large number of clinical specimens obtained from cases with various provisional diagnoses, all within a setting that had no restrictions on test ordering. Additionally, all cases had a final diagnosis based on both microbiological results and clinical responses to antifungal treatment. We acknowledge several limitations, including the retrospective, single-center design, which limits generalizability. The inclusion of a large proportion of cases with non-fungal final diagnoses skewed the overall impact metrics. We did not perform other targets such as the D1/D2 region of the 28S subunit, the β-tubulin gene, and EF-1α, which would better differentiate some fungi. Rarely, ITS heterogeneity has been described in pathogenic fungi, which has resulted in difficult interpretation of direct sequencing of the PCR product (21). Finally, the long TAT of ITS sequencing results in our setting relegates ITS to a less impactful role in acute management.

In summary, in an unrestricted clinical setting, the overall sensitivity of ITS sequencing for diagnosing fungal infection was low, and the direct clinical impact of ITS sequencing on antifungal management was small. The low impact was primarily exacerbated by a prolonged TAT for sequencing results. The utility of ITS sequencing is limited in BAL fluid due to a high proportion of colonization. To maximize the cost-effectiveness and clinical benefit of ITS sequencing, laboratory ordering restrictions should be implemented, and efforts should be made to reduce test result TAT.

## Data Availability

The data sets used and analyzed during the current study are available from the corresponding author upon reasonable request.

## SUPPLEMENTAL MATERIAL

Table S1 spectrum.03964-25-s0001.pdf Details of cases with ITS sequencing positive.

ASM does not own the copyrights to Supplemental Material that may be linked to, or accessed through, an article. The authors have granted ASM a non-exclusive, world-wide license to publish the Supplemental Material files. Please contact the corresponding author directly for reuse.
