# Supplementary material for: Diagnostic yield and impact of internal transcribed spacer sequencing from clinical specimens on antifungal management in patients with suspected fungal infection
Source: Microbiol Spectr. 2026 Sep 8;14(10):e03964-25. doi: 10.1128/spectrum.03964-25 (PMC13689364; doi:10.1128/spectrum.03964-25)
Supplement: Supplementary file 1 [file spectrum.03964-25-s0001.pdf]

Table S1 Details of cases with ITS sequencing positive

Specimen (no.)	Type of specimen	ITS sequencing identification	Fungal culture identification	Impact on management	Antifungal use at specimen collection	TAT (days)	Final diagnosis
33	BAL	*Candida albicans*	*Candida albicans*	No: long TAT (death)	No	10	*Stenotrophomonas maltophilia* pneumonia
49	BAL	*Aspergillus fumigatus*	*Candida albicans*	No: prior fungal antigen positive	No	10	Probable IPA
67	BAL	*Candida albicans*	*Candida albicans*	No: non-Infection/colonization	No	13	Multilobar pneumonia
78	BAL	*Cladosporium cladosporioides*	Not detected	No: non-Infection/colonization	29 days of voriconazole	12	Disseminated TB, Probable IPA
108	Skin tissue	*Cryptococcus neoformans*	*Cryptococcus neoformans*	No: continuation	No	15	Disseminated cryptococcosis
123	Brain tissue	*Chrysocorona lucknowensis*	Dermatiaceous fungi non-sporulate	Yes: adaptation	No	18	Dermatiaceous brain abscess
131	Sphenoid mucosa tissue	*Aspergillus fumigatus*	*Aspergillus fumigatus*	No: tissue/histopathology positive	No	13	Invasive aspergillus sinusitis
132	Ethimoid mucosa tissue	*Aspergillus fumigatus*	*Aspergillus fumigatus*	No: tissue/histopathology positive	No	13	Invasive aspergillus sinusitis
133	Sphenoid mucosa bone	*Aspergillus fumigatus*	*Aspergillus fumigatus*	No: tissue/histopathology positive	No	13	Invasive aspergillus sinusitis
134	Sphenoid mucosa tissue	*Aspergillus fumigatus*	*Aspergillus fumigatus*	No: tissue/histopathology positive	No	13	Invasive aspergillus sinusitis

135	Sphenoid mucosa tissue	*Aspergillus fumigatus*	*Aspergillus fumigatus*	No: tissue/histopathology positive	No	13	Invasive aspergillus sinusitis
136	Sphenoid mucosa tissue	*Aspergillus fumigatus*	*Aspergillus fumigatus*	No: tissue/histopathology positive	No	12	Invasive aspergillus sinusitis
137	Sphenoid mucosa tissue	*Aspergillus fumigatus*	*Aspergillus fumigatus*	No: tissue/histopathology positive	No	12	Invasive aspergillus sinusitis
164	BAL	*Candida glabata*	Not detected	No: non-Infection/Colonization	No	12	Probable IPA
196	BAL	*Candida nivariensis*	*Candida nivariensis* *Candida tropicalis*	No: microscopic examination positive	1 day of fluconazole	11	Candida CRBSI with pneumonia
197	BAL	*Candida nivariensis*	*Candida nivariensis* *Candida tropicalis*	No: microscopic examination positive	1 day of fluconazole	11	Candida CRBSI with pneumonia
206	Frontal sinus mucosa tissue	*Exserohilum* spp.	Not detected	No: microscopic examination positive	No	15	Invasive fungal sinusitis
216	BAL	*Candida dubliniensis*	*Candida dubliniensis*	No: non-infection/colonization	No	14	Necrotizing pneumonia
233	BAL	*Candida albicans*	Not detected	No: non-infection/colonization	No	15	Amiodarone induced pneumonitis
239	Cornea tissue	*Trichomonascus ciferrii*	*Penicillium* spp.	No: continuation	No	11	Mixed fungal and herpes keratitis
249	BAL	*Candida glabrata*	Terminated due to bacterial overgrowth	No: non-infection/colonization	No	13	Recurrent *Pseudomonas aeruginosa* pneumonia
261	Hip tissue	*Candida nivariensis*	Not detected	No: non-infection/colonization	No	16	Chronic osteomyelitis of the hip

266	BAL	*Candida albicans*	*Candida albicans*	No: prior culture positive	No	14	Candida CRBSI with pneumonia, Probable IPA
276	BAL	*Candida glabrata*	*Candida albicans*	No: non-infection/colonization	No	12	Proven IPA
280	BAL	*Aspergillus* spp.	*Candida albicans*	No: prior fungal antigen positive	No	12	Probable IPA
284	BAL	*Candida albicans*	*Candida albicans*	No: non-infection/colonization	No	10	Probable IPA
294	Arm tissue	*Cladosporium* spp.	Not detected	No: non-infection/colonization	No	24	Chronic infection of the forearm
296	BAL	*Candida albicans*	*Candida albicans*	No: non-infection/colonization	No	22	Ventilator associated pneumonia
297	Toe tissue	*Phaeoacremonium spp.*	*Phaeoacremonium* spp.	Yes: introduction	No	14	Cutaneous phaeohyphomycosis

Abbreviations: CRBSI: catheter-related bloodstream infection, IPA: invasive pulmonary aspergillosis, TB: tuberculosis, TAT: turnaround time
